# A low-cost simulation model for priapism detumescence training

**DOI:** 10.4102/jcmsa.v3i1.223

**Published:** 2025-07-30

**Authors:** Sachin Garach, Hanlie Dreyer, Ahmed Adam, Abdullah Laher

**Affiliations:** 1 Department of Emergency Medicine, Tambo Memorial Hospital, University of the Witwatersrand, Johannesburg, South Africa; 2 Department of Emergency Medicine, Faculty of Health Sciences, University of the Witwatersrand, Johannesburg, South Africa; 3 Division of Urology, Department of Surgery, Faculty of Health Sciences, University of the Witwatersrand, Johannesburg, South Africa

**Keywords:** ischaemic priapism, simulation training, needle aspiration, low-cost simulation model, detumescence, emergency medicine education, urological emergencies

## Abstract

Ischaemic priapism is a urological emergency requiring prompt intervention to prevent complications such as erectile dysfunction. Because of its low incidence, opportunities for hands-on training are limited, particularly in resource-constrained settings. To address this gap, we developed a low-cost, anatomically realistic simulation model to train emergency medicine and urology trainees in needle aspiration techniques. Constructed from readily available materials, the model replicates the anatomical and hepatic characteristics of priapism and was successfully integrated into a structured training programme for emergency medicine registrars. Although not formally evaluated, trainee feedback indicated improved procedural skill and confidence. The constructed reusable trainer offers an accessible and effective educational tool to enhance clinical preparedness, especially in low-resource environments. Further studies are needed to evaluate its impact on training outcomes.

## Introduction

Priapism is a pathological condition characterised by a persistent, often painful penile erection lasting more than 4 h, unrelated to sexual stimulation. It has an estimated incidence of 0.73 per 100 000 males in the United States.^1^ Priapism is classified into three types: ischaemic (low-flow), non-ischaemic (high-flow), and stuttering (recurrent). Among these, ischaemic priapism is the most common and clinically significant, constituting a urological emergency that requires prompt intervention to prevent irreversible tissue damage and long-term complications such as erectile dysfunction.^2^

A cornerstone of ischaemic priapism management is needle aspiration, a minimally invasive procedure in which a 19–21 gauge needle is inserted into the corpora cavernosa to aspirate deoxygenated blood. The procedure is typically performed under local anaesthesia and guided by palpation or imaging to optimise accuracy and reduce patient discomfort. If aspiration alone is insufficient, intracavernosal injection of sympathomimetic agents such as phenylephrine is often employed.^3^

Despite the effectiveness of current interventions, opportunities for hands-on training in priapism management remain limited, particularly in low-resource settings. Given the condition’s rarity and the need for urgent intervention, simulation-based education is essential to ensure procedural competence and clinician confidence. Simulation-based training using a priapism model has been shown to provide a valuable educational experience.^4^ This article outlines the design, construction, and implementation of a low-cost, anatomically realistic priapism trainer aimed at supporting skill development in needle aspiration for ischaemic priapism, particularly within emergency medicine and urology training programmes.

## Materials and model construction

A low-cost, anatomically realistic simulation model was developed using readily available materials, making it particularly suitable for use in resource-limited training environments. Materials were sourced from a local hardware store, pharmacy, and adult store. The design builds upon previously described models,^5,6^ incorporating several novel modifications to enhance anatomical realism, haptic feedback, and durability.

The model consists of a penis sleeve filled with Styrofoam to simulate the consistency of soft tissue. A Penrose drain or heat-shrink tubing filled with Betadine solution is embedded within the sleeve to represent the blood-filled corpora cavernosa. This configuration offers realistic tactile fidelity and supports repeated use for procedural training. While the current design effectively simulates both corpora cavernosa, it does not include a urethral component. Future iterations could enhance anatomical fidelity by incorporating an 18Fr Silastic catheter to simulate the urethra, thereby further improving the model’s realism for training purposes.

### Materials

**FIGURE 1:**
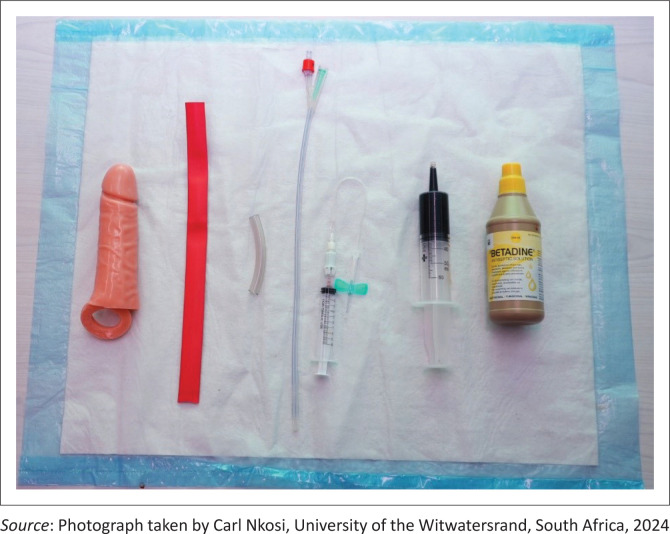
Materials used in the construction of the low-cost priapism simulation model.

Materials used to develop the simulation model (see Figure 1):

- Penis sleeve
- Penrose drain or heat-shrink tubing
- Styrofoam
- Hot glue
- Foley urinary catheter
- Betadine solution
- Butterfly needle (18G/20G)
- Gelatine and water

### Model construction

The model was constructed as follows:

- The hollow penis sleeve is packed with a Penrose drain (or heat-shrink tubing) surrounded by Styrofoam to simulate erectile tissue.
- One end of the tubing is sealed with hot glue, while the other is connected to a Foley catheter with an inflated bulb.
- The interior is filled with a gelatine mixture to ensure structural rigidity.
- Betadine is introduced via the catheter to simulate pooled deoxygenated blood.
- Fluid is aspirated using a butterfly needle to simulate the clinical procedure.

## Cost analysis

The total cost of constructing one simulation model is approximately ZAR445.00 in Johannesburg, South Africa, equivalent to $25.13 based on the current exchange rate ($1 = ZAR 17.71). A breakdown of the individual material costs is provided below:

- Penrose drain or heat-shrink tubing – ZAR20.00 (~$1.13)
- Hot glue – ZAR50.00 (~$2.82)
- Betadine – ZAR35.00 (~$1.98)
- Butterfly needle – ZAR30.00 (~$1.69)
- 20 mL syringe – ZAR10.00 (~$0.56)
- Foley catheter – ZAR150.00 (~$8.47)
- Penis sleeve – ZAR150.00 (~$8.47)

## Implementation and training application

The model is reusable with proper technique, including reinjection of Betadine solution into the simulated corpora cavernosa between uses. Refrigeration is recommended to preserve the solution’s viscosity and enhance durability.

**FIGURE 2:**
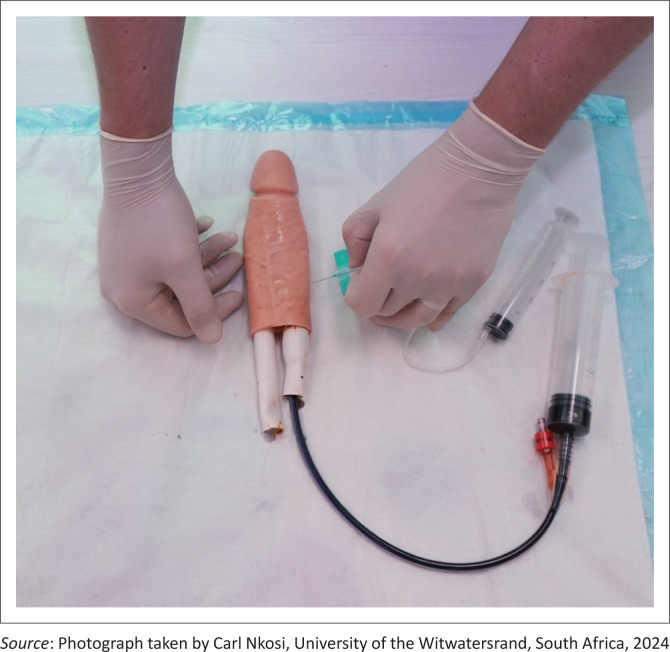
Illustration of the needle aspiration site within the ‘corpus cavernosum’ of the priapism simulation model.

It was integrated into a structured emergency medicine training module, beginning with a brief didactic session on the pathophysiology and management of priapism, followed by hands-on practice of needle aspiration using a butterfly needle (Figure 2).

The model proved to be a cost-effective and practical tool for training in priapism detumescence. While not formally evaluated, trainee feedback consistently indicated improved skill and confidence in performing the procedure. By simulating a high-stakes, time-sensitive intervention, the model supports the development of procedural competence and decision-making. Standardised use across training programmes may improve consistency and preparedness.

Ongoing simulation-based assessments can help monitor competency progression. Future studies should evaluate the model’s impact on clinical performance and patient outcomes.

## Conclusion

This low-cost, anatomically realistic simulation model provides an effective and accessible tool for training healthcare providers in the management of ischaemic priapism. It facilitates hands-on practice of needle aspiration in a safe and controlled setting, thereby enhancing procedural confidence and clinical readiness. Given the critical importance of timely intervention, such training tools may be especially valuable in resource-limited environments, where they can help address gaps in clinical preparedness. Further research is warranted to evaluate their impact on clinical performance and patient outcomes.
